# Circulating bile acids concentration is predictive of coronary artery disease in human

**DOI:** 10.1038/s41598-021-02144-y

**Published:** 2021-11-22

**Authors:** Caroline Chong Nguyen, Denis Duboc, Dominique Rainteau, Harry Sokol, Lydie Humbert, Philippe Seksik, Adèle Bellino, Hendy Abdoul, Naïm Bouazza, Jean-Marc Treluyer, Malika Saadi, Karim Wahbi, Heithem Soliman, Benoit Coffin, André Bado, Maude Le Gall, Olivier Varenne, Henri Duboc

**Affiliations:** 1 Centre de Recherche Sur I’inflammation, Inserm, UMR 1149, Université de Paris, 75018 Paris, France; 2 Cardiology Department, Cochin Hospital, Assistance Publique–Hôpitaux de Paris, Université de Paris, 75014 Paris, France; 3 Department of Hepato Gastro Enterology and University of Paris, Louis Mourier Hospital, APHP, 92700 Colombes, France; 4 Inserm, Centre de Recherche Saint-Antoine, CRSA, AP-HP, Hôpital Saint Antoine, Service de Gastroentérologie, Sorbonne Université, 75012 Paris, France; 5 Unite de Recherche Clinique-Centre Dinvestigation Clinique Necker/Cochin, Hôpital Tarnier, Université de Paris, 75006 Paris, France; 6 INSERM UMRS 1149, Université de Paris, 16 rue Henri Huchard, 75890 Paris Cedex 18, France

**Keywords:** Gastroenterology, Cardiovascular diseases

## Abstract

Synthetized by the liver and metabolized by the gut microbiota, BA are involved in metabolic liver diseases that are associated with cardiovascular disorders. Animal models of atheroma documented a powerful anti-atherosclerotic effect of bile acids (BA). This prospective study examined whether variations in circulating BA are predictive of coronary artery disease (CAD) in human. Consecutive patients undergoing coronary angiography were enrolled. Circulating and fecal BA were measured by high pressure liquid chromatography and tandem mass spectrometry. Of 406 screened patients, 80 were prospectively included and divided in two groups with (*n* = 45) and without (*n* = 35) CAD. The mean serum concentration of total BA was twice lower in patients with, versus without CAD (*P* = 0.005). Adjusted for gender and age, this decrease was an independent predictor of CAD. In a subgroup of 17 patients, statin therapy doubled the serum BA concentration. Decreased serum concentrations of BA were predictors of CAD in humans. A subgroup analysis showed a possible correction by statins. With respect to the anti-atherosclerotic effect of BA in animal models, and their role in human lipid metabolism, this study describe a new metabolic disturbance associated to CAD in human.

## Introduction

Cardiovascular diseases (CVD) remain a major cause of death, whose prevention of modifiable risk factors can lower the incidence of ischemic events^1,2^. Increased morbidity and mortality by CVD are undoubtedly observed during Non Alcoholic Fatty Liver Disease (NAFLD)^3^, supported by overlapping risk factors. Beyond the clinical association, fundamental research describes numerous mechanistic axes that are shared between CVD and NAFLD, including disorders of lipid-glucid metabolism, systemic and local inflammation, and fibrosis^4,5^. In all these themes, BA have been associated in the literature, either with a protective^6–8^ or a worsening^9–11^ role in these processes.

Acting as hormones through the vascular system, BA are “wandering molecules” which regulate the metabolism and inflammation in metabolic disorders^12^: the permanent loop between their secretion in the gut lumen, their microbiota transformation, their ileal reabsorption and their vascular transportation through the portal blood to the liver—known as the entero-hepatic cycle- is a bridge between the microbiota and the host. The synthesis by the liver from cholesterol under the primary cholic (CA) and chenodeoxycholic acid (CDCA) forms is the first metabolic step^13^. Once conjugated to taurin or glycin, they are stocked in bile. Upon postprandial emptying of the gallbladder, they reach the lumen and promote the micellization of alimentary fat. In the gut, they are metabolized by the microbiota, consisting of a bacterial deconjugation followed by a transformation (dehydroxylation), leading to the secondary deoxycholic acid (DCA) and lithocholic acid (LCA)^13^. By modifying the stereospatial configuration of BA, the microbiota directly influences the composition of the bile acids pool^13^, as well as their binding and activation properties to their specific receptors^14,15^. This loop is closed when 95% of the BA are reabsorbed in the ileum, returning to the liver via the portal circulation. In the peripheral blood, BA circulate instable concentrations, with a prominent postprandial peak (the “spill over”) following their rapid reabsorption by the gut^16^.

The two main BA receptors are the membrane TGR5^17^ and the nuclear FXR^18^. Both are implicated in NAFLD and in CAD pathogenesis. Obeticholic acid, a BA derivative, is a dual agonist of these receptors, whose use in humans has shown a histological benefit in NASH patients in liver biopsies^19^. In LDL -/- mice, the activation of TGR5 slowed the development of atheroma by targeting the macrophages in the plaque, decreasing the production of pro-inflammatory cytokines and inhibiting the oxidized LDL uptake by lowering the expression of CD36^8^. A similar protective role has also been attributed to FXR. On a high fat diet, the plaque development is 50% greater in double KO LDL-/- and FXR -/-, than in simple KO LDL -/-mice, with similar effects observed in ApoE -/- mice^20,21^. Of note, atherosclerosis is more than a mere aging process, implying the participations of pro-inflammatory and metabolic disorders^1^: several studies in animals and humans have linked the stimulation of BA receptors to other beneficial metabolic effects, in energy expenditure^22^ or glucido-lipid metabolism^6^.

In order to specifically address the question of the clinical relevance of CAD and BA in human, the main objective of our study was to compare the BA pool of patients with versus without angiographically confirmed coronary artery disease (CAD). The microbiota being a determining factor in the qualitative composition of the BA pool, the second objective was to compare the microbiota composition of the two groups discriminated by coronary angiograms. Applying rigorous inclusion criteria to prevent variations in the microbiota and BA metabolism^23^, we measured the serum and fecal BA concentrations and composition. We estimated the daily liver synthesis by measuring 7-alpha C4, a precursor molecule^24^, and the microbiota composition by 454 pyrosequencing.

## Results

### Study population

**Figure 1:**
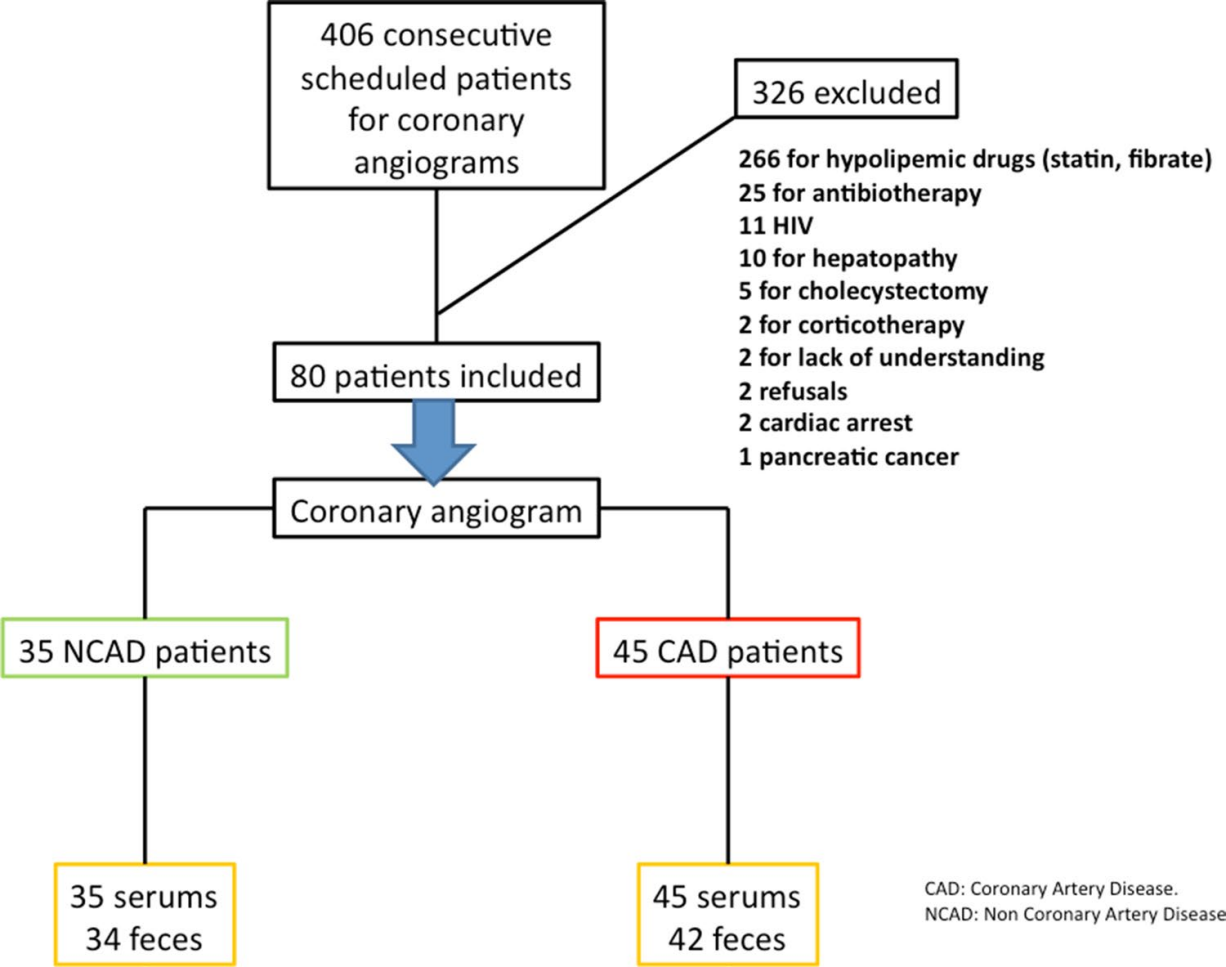
Flow chart of patients screened, excluded and enrolled in each study group, and their contributions of serum and fecal samples.

Between February and May 2015, 406 patients were hospitalized to undergo a scheduled coronary angiogram, of whom 326 were excluded for various criteria (Fig. 1). The indications for coronary angiogram and the disease severity are shown in supplementary table 2. Ultimately, 80 patients were included in the study, of whom 45 presented with significant CAD and 35 were normal. The clinical characteristics of the two groups are detailed in Table 1. They were comparable except for 3 characteristics: age, gender and diastolic blood pressure. The group presenting with CAD was significantly older, more often men, and had a higher diastolic blood pressure.

**Table 1:** Clinical characteristics of patients with and without coronary artery disease.

	NCAD (*N* = 35)	CAD (*N* = 45)	*P*
Demographic characteristics			
Male gender	17 (49%)	33 (73%)	*P* = 0.02
Age	57 ± 2.3	66 ± 1.3	*P* = 0.004
Cardiovascular risk factor			
Weight (kg0	74 ± 2.8	77 ± 1.7	*P* = 0.28
Height (m)	1.7 ± 0.02	1.72 ± 0.01	*P* = 0.20
BMI (kg/m^2^)	26 ± 0.75	25.9 ± 0.5	*P* = 0.64
Hypertension	10 (29%)	21 (47%)	*P* = 0.11
Systolic blood pressure (mmHg)	132 ± 3.6	138 ± 3.7	*P* = 0.21
Diastolic blood pressure (mmHg)	72 ± 2.2	77 ± 1.8	*P* = 0.04
Smoke	16 (46%)	22 (49%)	*P* = 0.82
Dyslipidemia	1 (3%)	5(11%)	*P* = 0.22
Diabetes	6 (17%)	8 (18%)	*P* = 1
Hereditary	6 (17%)	9 (20%)	*P* = 0.78
Biological results			
Total cholesterol (g/l)	2 ± 0.17	2 ± 0.17	*P* = 0.42
Triglycerides (g/l)	1.2 ± 0.1	1.3 ± 0.11	*P* = 0.09
HDL (g/l)	0.6 ± 0.05	0.6 ± 0.05	*P* = 0.36
LDL (g/l)	0.9 ± 0.09	1.15 ± 0.10	*P* = 0.53
Creatinine level (μmol/l)	85 ± 10	81 ± 3.2	*P* = 0.17

NCAD: No Coronary Artery Disease. CAD: Coronary Artery Disease.

### Bile acids deficiency in sera is predictive of CAD

**Figure 2:**
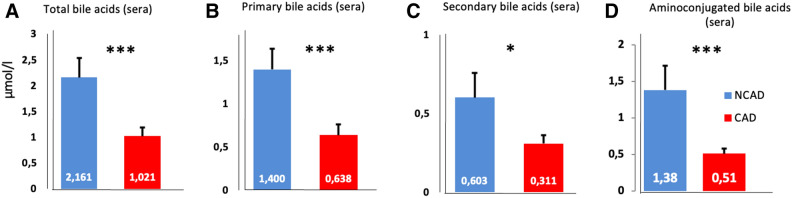
Comparison of bile acids concentrations in sera in non-coronary artery disease (blue bars) and coronary artery disease (red bars) patients. Concentrations (µmol/l) of total (**A**), primary (**B**), secondary (**C**) and amino-conjugated (**D**) bile acids in sera. Image credit: Graphpad 6.0, https://www.graphpad.com/scientific-software/prism/.

The total concentration of BA in sera (the sum of the 28 BA species) was two-fold lower in patients with than without CAD (Fig. 2A). By multiple variable analysis, the total BA concentration, adjusted for age and gender, was highly predictive of CAD. The area under the curve (AUC) was 83.6% (74.5–92.8%), and OR (95%CI) was 0.51 (0.31, 0.85), *P* = 0.01 (Fig. 4A; supplementary table 3).

The primary, secondary, and conjugated serum BA concentrations were decreased and systematically lower in patients with, than in patients without CAD (Figs. 2B–D). However, the proportions of primary, secondary or conjugated BA were similar, with a global decrease in BA species in patients presenting with CAD (supplementary table 4B).

We therefore sought to identify a candidate bile acid that could be a single marker reflecting the overall decline in the 28 circulating bile acid species. We oriented our analysis towards G-CDA, described in the literature as the primary bile acid synthesized most abundantly by the liver in comparison with other primary bile acids. Among the 28 BA isolated in sera, the glyco-conjugated GCDCA acid was the most abundant, representing 30% of all BA species, (supplementary figure 2). This proportion was similar in both groups (data not shown). Like total BA, the concentration of GCDCA was twofold lower in patients with than in patients without CAD. The serum GCDCA concentration was similarly predictive of CAD, with an AUC of 83.6% (74.1–93.0%) and OR of 0.06 95% CI 0.01, 0.51; *P* = 0.01 (Fig. 4B; supplementary table 3).

### Unchanged concentrations and composition of BA in feces of patients presenting with CAD

**Figure 3:**
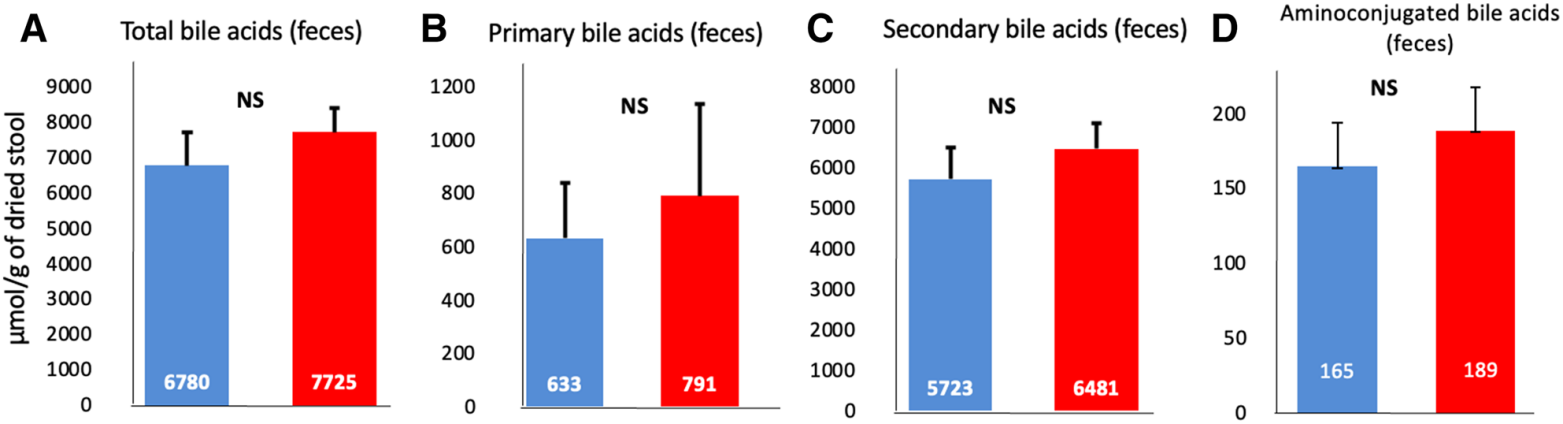
Comparison of bile acids concentrations in feces in non-coronary artery disease (blue bars) and coronary artery disease (red bars) patients. Concentrations (µmol/g of dried stools) of total (**A**), primary (**B**), secondary (**C**) and amino-conjugated (**D**) bile acids in feces. *P* < 0.05; **P < 0.01; ***P < 0.001. Image credit : Graphpad 6.0, https://www.graphpad.com/scientific-software/prism/.

In contrast to the sera, the total concentrations of BA in feces samples were similar in both study samples (Fig. 3A). The main, primary, secondary, and conjugated species were also similar, in concentrations as well as proportions (Figs. 3B–D, and supplementary table 5).

### Similar hepatic bile acids synthesis in patients with and without CAD

**Figure 4:**
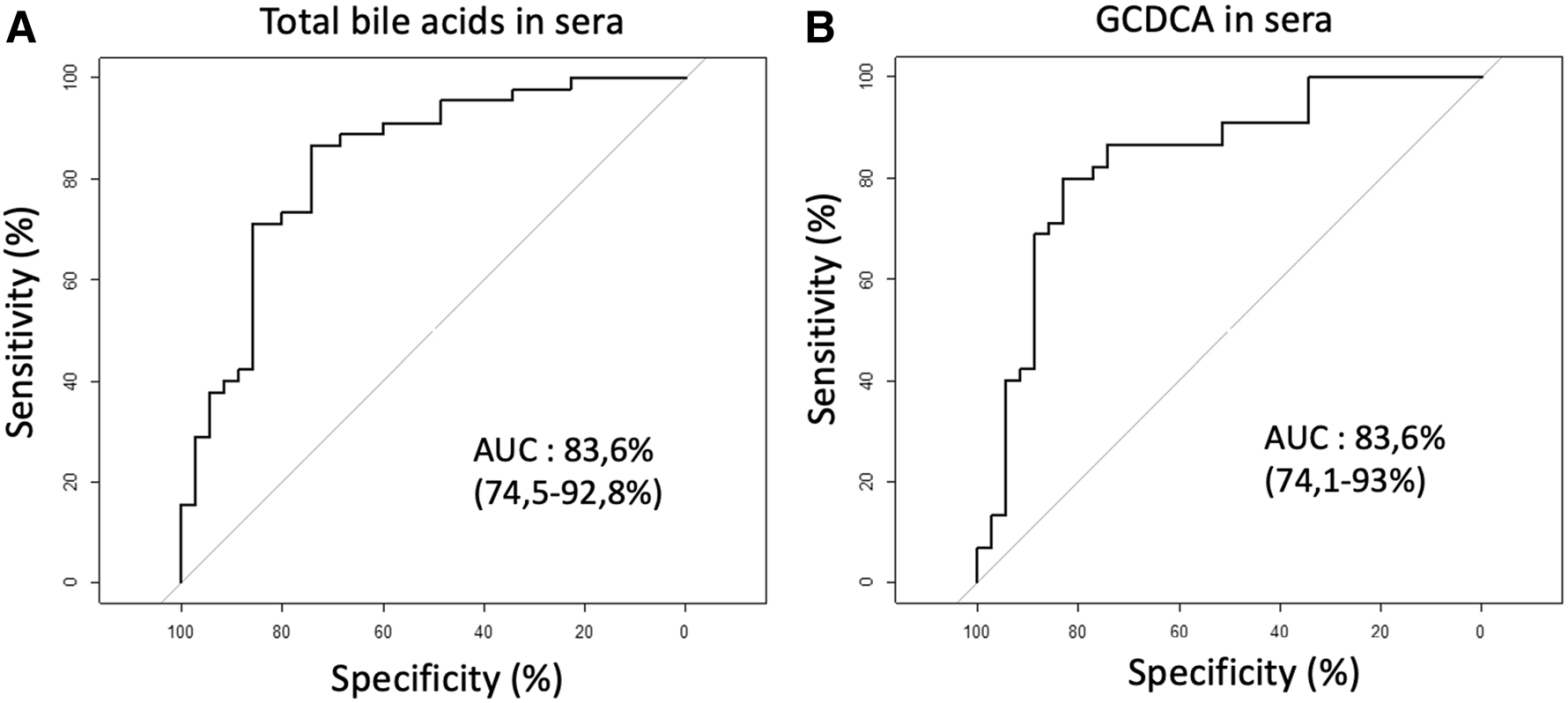
Receiver operating characteristic curves using as predictive factors of coronary artery disease adjusted for age and gender: (**A**) total bile acids concentrations in sera; (**B**) glycho-chenodeoxycholic acid (GCDCA) in sera. Image credit: R software: https://www.r-project.org.

**Figure 5:**
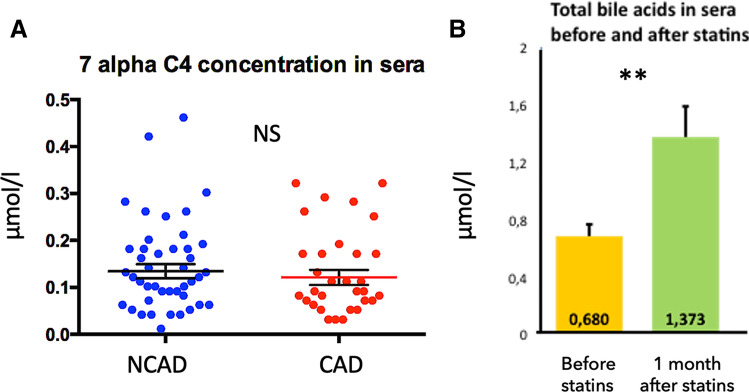
(**A**) Concentrations (µmol/l) of 7 alpha C4, a liver metabolite reflecting the daily hepatic bile acid synthesis in sera in the two study samples. (**B**) Comparison of total bile acids concentrations in a subset of 17 patients with coronary artery disease after 1 month of statin therapy. NCAD = non-coronary artery disease; CAD: = coronary artery disease. **P < 0.01. Image credit: Graphpad 6.0, https://www.graphpad.com/scientific-software/prism/.

The measurement of 7alpha C4 in serum is an indirect reflection of the total daily hepatic synthesis of BA^24^. This metabolite is the first step of hepatic production of bile acids from cholesterol. It's produced by CYP7A1 enzyme, which is the rate-limiting enzyme in bile acid synthesis, and whose enzymatic activity measured on liver biopsy is directly correlated to the circulating levels of 7α-hydroxy-4-cholesten-3-one^25^. The concentrations of 7alpha C4 in sera was similar in the two groups (0.13 ± 0.01 vs 0.12 ± 0.01 μmol/l; Figs. 4, 5A).

### Significant increase in the concentrations of bile acids by statins in the serum of patients with CAD

In the subset of 17 patients with CAD, the mean concentration of total BA doubled after 1 month of statin therapy, from 0.68 ± 0.08 to 1.37 ± 0.21 μmol/l, *P* = 0.01; Fig. 5B). Total BA concentration increased in 13 of 17 patients (77%) and decreased in 4 of 17 patients (23%).

The supplementary figure 3 shows the individual evolution of total serum bile acid concentrations after one month in each of the 17 patients.

### The fecal microbiota composition is not predictive or specific of CAD

**Figure 6:**
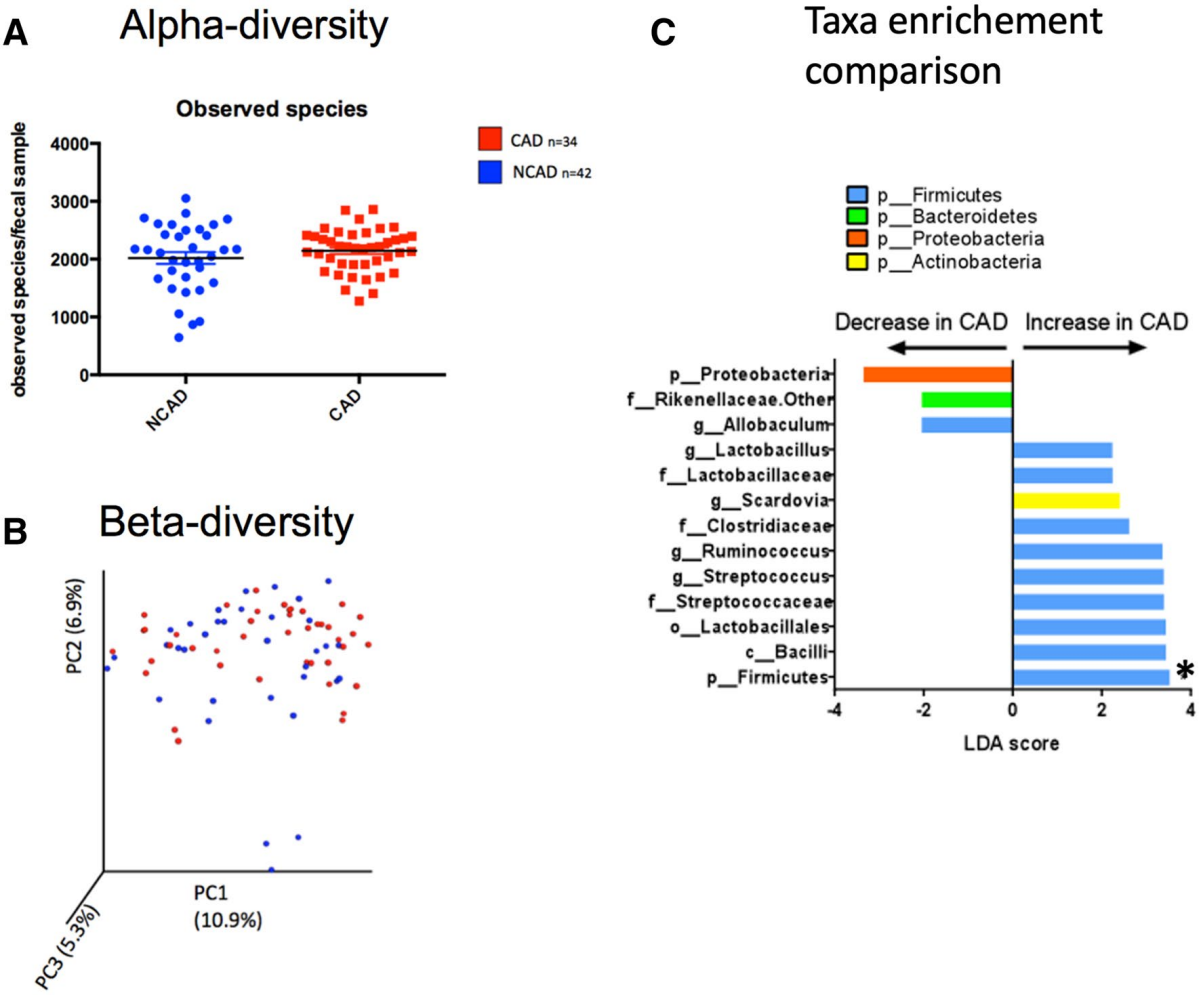
Descriptive and comparative analysis of the fecal microbiota in both patient samples: (**A**) Intra sample bacterial diversity, represented by the alpha diversity index, and illustrated by the number of bacterial species observed in each fecal sample. (**B**) Inter sample bacterial diversity, represented by the beta diversity index, illustrated by the Bray Curtis distance analyzed by primary component analysis. (**C**) Bacterial taxa enrichment in patients with coronary artery disease using LeFSE analysis.* Taxa significant after adjustment for age and gender, using a multiple variable association with linear models. NCAD = Non-coronary artery disease; CAD = Coronary artery disease. Image credit: Figure **A** and (**B**): Graphpad 6.0, https://www.graphpad.com/scientific-software/prism/. Figure (**C**): LEfsE, https://huttenhower.sph.harvard.edu/lefse/.

The richness of fecal microbiota was similar in both groups. The overall number of species observed in a sample, represented by the alpha diversity (Fig. 6A) and other alpha diversity, represented with the Shannon, Simpson, and Chao-1 indexes (supplementary figure 1) were similar. The beta (inter-sample) diversity, represented by the Bray Curtis index, was also unspecific in patients with versus without CAD (Fig. 6B). While no bacterial clusters in the microbiota composition was identified in any of the groups, the firmicutes phylum at the taxa levels were more prominently represented in the feces of patients with CAD (Fig. 6C).

## Discussion

The objective of this work was to determine in humans whether there are observable qualitative or quantitative differences in circulating and fecal bile acids, and in the composition of the microbiota during CAD. We used strictly controlled sampling conditions in a carefully recruited population, justifying the 80% of non-inclusion of eligible patients, to avoid artificial variations induced by multiple factors that may influence the determination of circulating bile acids^26^. In a comparison of patients with and without CAD, we observed in patient with CAD a quantitative defect affecting all species of circulating bile acids, with an overall concentration halved. We identified GCDCA, a unique BA among the 28 acids, as a marker of this defect, and a predictive factor of CAD. We also observed that this decrease in BA could be corrected by the administration of statins. We observed no specific pattern nor in the qualitative repartitions of BA pool, neither composition of the fecal microbiota.

This work has some limitations: the first one is the identification of patients with versus without atheroma based on coronary angiograms. Since it reveals the presence of intraluminal arterial irregularities, coronary angiograms, as opposed to computed tomography scanning, may miss extra luminal atheromas. We chose coronary angiograms when designing the study, as our patients would not have systematically undergone scanning, whereas they all had a scheduled angiogram before their inclusion. Second, patients with CAD were older than patients without, as if it was not a surprising observation—aging being a risk factor for cardiovascular events. Moreover, our selective screening aimed at eliminating confounding factors : we can then consider that a weakness of the study is that NASH / NAFLD pathologies were not screened in the patient population, by non-invasive tests (liver elastometry combined with blood tests)^27^. Abnormalities of bile acid metabolism have been described in these metabolic pathologies^28^, which are associated with the development of atheroma. However, there was no difference between the two groups in terms of the associated co-morbidities that prompt the search of a NAFLD (obesity, metabolic syndrome, diabetes)^29^ and patients with alteration of liver enzymes were systematically excluded.

These observations support the hypothesis that BA could acts as natural braking mechanism, protecting against the development of atheromatous plaques. So far, in human the literature is scarce. Recently, Li et al.^30^ observed similar results in a cohort of more than 7400 patients, associating a decrease in circulating total bile acids with the presence, and also the severity, of CAD. Although the exclusion criteria did not include statin use, and although the enzymatic method used to measure bile acids is less accurate than HPLC MS/MS, it is interesting to note that the decrease in circulating bile acids could be observed in two prospective studies of large and small effective, that methodologically segregated two populations on coronary angiography data.

Our study does not provide any mechanistic approach for this observation. Through the literature, two different mechanistic axes supporting an anti-atherogenic effect of bile acids are proposed. Firstly, a molecular action of bile acids by their TGR5 and FXR receptors, driving their anti-inflammatory effect in vitro^31,32^, as well as several beneficial actions on the metabolism in vivo^6^. Recent evidences that BA acts as hormones supports this molecular action, targeting their receptors through the serum with metabolic effect in various tissues^33^. The experimental observation of protection against atheroma comes mainly from the literature in animal models^8,20,21,34,35^. Drugs targeting the BA receptors, such as obeticholic acid, had also promising effects in NAFLD, that is clinically associated to CVD ^19,36^. But to date, no effect of a bile acid receptor-targeting drug is described in CAD.

Secondly, the protective effect could be linked to a quantitative evacuation of BA, through their fecal excretion. The induction of an intestinal loss of BA after binding to BA sequestrants (colesevelam or cholestyramin) is a known pathway of cholesterol elimination through the gut, that can lower the concentration of LDL. ^37,38^ Decreasing the BA pool, triggers a reaction in the liver, that promotes the conversion of the intracellular cholesterol to BA synthesis: colesevelam and cholestyramine could increase the circulating concentration of cholic acid, whereas the total BA concentrations remain stable or decrease^39,40^. Despite encouraging studies, the ability of bile acids sequestrants to slow the progression of CAD remains uncertain^41^.

Routinely prescribed after a cardiovascular event, lipid lowering statins therapy reduces in morbidity and mortality in CVD^42^. It also have been proven to be safe and possibly effective in the treatment of NAFLD/NASH^43–45^. Interestingly, we also observed that 1 month of statin therapy doubled the concentrations of circulating BA in patients with CAD. This subset of 17 patients began statin therapy for secondary prevention of CAD (16 patients received 10 to 80 mg and 11 patients received 40 mg of atorvastatin daily). In mice, atorvastatin causes an over expression of CYP7A1, the liver enzyme responsible of BA synthesis, and changes the BA pool composition^46^, without, however, increasing the pool of circulating BA. It is noteworthy that 17 of these 17 patients also received antiplatelets agents, 13 received a beta-adrenergic blocker, and 13 received an angiotensin converting-enzyme inhibitor. The increase in total BA might also be theoretically attributed to these other drugs, even though unlike statins, they do not target the cholesterol metabolism.

We have not described the mechanism explaining this decrease in the concentrations of circulating BA. With regard to the similar concentrations of 7alpha C4 ^24^ in the sera, the lower concentrations of BA in patients suffering from CAD is probably not the consequence of a lower synthesis (this metabolite is an indirect marker of the daily hepatic synthesis of BA^24,25^). Similarly, the similar fecal BA concentrations suggest that the intestinal excretion is unchanged in both groups. However, a spot sample is insufficient to measure the true excretion of BA, which requires a 24 to 48 h stool collection. Other studies have confirmed that the 24-h intestinal excretion of BA is lower in patients with than in patients without CAD ^47^. As if this lower excretion has been correlated with stroke and mortality^48^, these studies did no measure BA in serum or its synthesis, to support the hypothesis of low BA producer patients in CAD. We hypothesize a mechanism that causes variations in the systemic passage of BA allowed by the liver, from the enterohepatic to the general circulation. The supplementary Figure 4 summarize the main results of this work. It proposes a "spill over" mechanism of BA: in the same way of glycemic peak after the meal, this postprandial spillover of BA into the general circulation, has been proposed as an important metabolic factor to take into consideration^16,49^.

One of our initial hypotheses was that the microbiota unbalance observed in patients presenting with atheroma, might change the BA metabolism. We found neither changes in the microbiota diversity, nor in the BA composition or their fecal concentration. The variation at the taxa level we observed cannot be considered a specific or a mechanistic marker^50^. The composition of the microbiota is not a mean of evaluating its function, and it is clear that some metabolites of the intestinal microbiota, such as trimethylamine, a derivative of L-carnitine transformed in the liver to trimethylamine-N-oxide (TMAO), increase the risk of cardiovascular events^51^. The TMAO measurement could have been adressed in that work. Of note, administration of TMAO in mice alters bile acids profiles and lowers hepatic bile acids synthesis, by reducing Cyp7a1 enzyme expression^52^.

Compositional differences in the gut microbiota were frequently reported in patient with CVD^53^ while we have not documented any specific one in this work. Methodologic differences^54^ make comparison with our results is problematic. Differences in the composition of the microbiota have been reported in a comparison of 12 patients with symptomatic carotid atheromas and 13 controls. In addition to the anatomical location, the extent and size of pathological atheromatous plaques is also very different in peripheral atheroma (carotid or femoral) compared to coronary atheromatous, so patients may not be comparable. Another study, using the same pyrosequencing technique in 15 patients presenting with atherosclerotic disease and in 15 healthy subjects, found no differences in the composition of the fecal microbiota^55^.

We propose here GCDCA as a marker of global BA deficiency observed in CAD. This primary gluco-conjugated BA is the most abundantly synthetized by the liver in human^26^. We observed that its concentrations vary similarly in patients and in controls, and were similarly predictive of CAD than the total concentrations of BA (Fig. 4B and supplementary figure 2). This point is interesting because it could dispense with the determination of 28 bile acids to observe the decrease of bile acids in case of CAD, by dosing only the GCDCA in routine.

In conclusion, our study identified a decrease in the concentrations of circulating BA as a predictor of atheroma visible on coronary angiograms, in absence of difference in the fecal BA concentration or composition, or in the fecal microbiota diversity. This deficiency could be correctd by statins administration. Our observation in highly selected patients, confirms the results of other recent work in humans^30^, and points to the liver rather than to the gut, as the BA metabolism is tightly linked to the cholesterol metabolism (Supplementary figure 4). Regarding the experimental protective properties of BA, further studies are needed to decipher how this observation fit into the complex pathophysiological molecular mechanisms of coronary atherogenesis.

## Methods

### Patients

This study was conducted in accordance with the guidelines of the Helsinki declaration, defining the ETHICAL PRINCIPLES FOR MEDICAL RESEARCH INVOLVING HUMAN SUBJECTS, (2013). The work was reviewed and approved by an institutional ethic comitee: COMITE DE PROTECTION DES PERSONNES ILE DE FRANCE III (registration number S.C. 3218, 2015). All patients granted their written informed consent to participate. This work has been registered in ClinicalTrials and entitled "Bile Acids and Gut Microbiota as Potent Coronary Atheroma Risk Factors: a Prospective Study in Human (MABAC, ClinicalTrials.gov Identifier: NCT02375893).

All patients aged from 18 to 79 years old included between February and May 2015 underwent coronary angiograms. In the morning of angiogram, venous blood and sampling of feces were collected in the fasting state. After immediate centrifugation, the sera were frozen at -80° C for later analysis. The fecal samples were immediately homogenized and frozen at -80° C. Patients in the non-fasting state or treated with statins or fibrates, corticosteroids, anti-human immunodeficiency virus agents, antimicrobials in the past 3 months, or suffering from liver disease or cancer, or who had experienced a recent cardiac arrest, or had undergone cholecystectomy, were excluded from this study.

Based on the coronary angiographic observations, the patients were divided between a group with or without coronary artery disease (CAD), regardless of its severity. In a subset of patients with CAD followed by our institution, we measured the quantitative effect of 1 month of statin therapy on BA in serum.

### Bile acids measurements in blood and feces

BA were measured in sera and feces, as previously described^56^, and detailed in the supplementary methods (supplementary table 1). Briefly, the BA were purified by solid phase extraction and measured, using a QTRAP® 2000, high-pressure liquid chromatography and tandem mass spectrometry system (Applied Biosystems/MDS SCIEX, Concord, Ontario, Canada**)**. Each peak was identified by comparing the spectrum of a range including 28 species of BA. The data were acquired and measured using the Analyst V.1.4.2 software (AB-SCIEX). The BA measurements were expressed in concentrations or as the percentage of each specific BA (± SEM) out of all BA after calibration of the method, with weighed mixtures and normalization relative to the internal standard (23-nor-5β-cholanoic acid-3α, 12α-diol). For the analysis, the BA were grouped in categories (supplementary table 1) or expressed individually. To estimate the liver synthesis of BA, we measured the 7a-hydroxy-4-cholesten-3-one (7a-C4) concentrations in serum, the first specific metabolite produced by the liver during hepatic synthesis, the plasma concentration of which reflects the daily rate of bile acid synthesis by the liver in man^24^. The C4 extraction method was similar to the bile acids extraction and the C4 quantification was performed as previously described, using a deuterated C4 form as internal standard (7α-hydroxy-4-cholesten-3-one-d7^49^.

### Fecal microbiota and diversity analysis by 454 pyrosequencing method

The fecal bacterial diversity was studied as previously described^57^, and is detailed in the supplementary methods. Briefly, total fecal DNA was extracted. The bacterial diversity was determined in each sample by targeting part of the ribosomal genes. The bacterial species were identified by sequencing the 16sRNA gene V3-V4 hypervariable regions. Richness of the samples were summarized by standard alpha diversity indexes, representing the intra-diversity of each sample (number of bacterial species observed, Shannon, Simpson and Chao1 index). The inter-sample diversity was represented by the Bray Curtis beta diversity index (principal component analysis of the Bray—Curtis distance). At the taxa classification level, the enrichment was compared using LEfSE analysis^17^, after adjustment for age and gender, using multivariate association with linear models.

### Statistical analysis

A descriptive analysis of the clinical and laboratory characteristics of the patient population was performed. Quantitative data are expressed as means ± standard deviation (SD) or median with interquartile range (IQR), and qualitative data as percentages with 95% confidence intervals (CI). Correlations between clinical and laboratory data were examined, using statistical tests appropriate for the variables studied. Mean values were compared by Student’s *t*-test or, if more appropriate, by Wilcoxon’s test. Percentages were compared, using the chi-square test of Pearson 2, or by Fisher’s exact test, if more appropriate. Logistic regression analyses were performed to examine the correlations between CAD and BA concentrations. Variables associated with CAD or its known risk factors, including hypertension, smoking, hyperlipidemia and diabetes, which emerged with a < 0.15 degree of significance by single variable analysis, were entered in a multiple variable regression analysis. Odd ratios (OR) and their 95% CI were calculated. All tests were two-sided with a 5% significance level. The analyses were performed using the R software, https://www.r-project.org. From the statistical results obtained on the R software, some graphical figures were constructed using graphpad 6.0, https://www.graphpad.com/scientific-software/prism/. The specific analysis of the gut microbiota is detailed in the supplementary methods.

## Supplementary Information

Supplementary Information.
