# Supplementary material for: Circulating bile acids concentration is predictive of coronary artery disease in human
Source: Sci Rep. 2021 Nov 22;11:22661. doi: 10.1038/s41598-021-02144-y (PMC8608912; doi:10.1038/s41598-021-02144-y)
Supplement: Supplementary file 1 — Supplementary Information. [file 41598_2021_2144_MOESM1_ESM.docx]

**Supplementary table 1: List of the 28 bile acids species measured in sera or feces**

| **Primary bile acids** | **Secondary bile acids** | **Ursodeoxycholic acids** |
| --- | --- | --- |
| **Cholic acid** | **Deoxycholic acid** | **Ursodeoxycholic acid** |
| **Tauro-Cholic acid** | **Tauro-Deoxycholic acid** | **Tauro-Ursodeoxycholic acid** |
| **Glyco-Cholic acid** | **Glyco-Deoxycholic acid** | **Glyco-Ursodeoxycholic acid** |
| **Cholic acid-3S** | **Deoxycholic acid-3S** | **Ursodeoxycholic acid-3S** |
| **Chenodeoxycholic acid** | **Lithocholic acid** | **Tauro-Ursodeoxycholic acid-3S** |
| **Tauro-Chenodeoxycholic acid** | **Tauro-Lithocholic acid** | **Glyco-Ursodeoxycholic acid-3S** |
| **Glyco-Chenodeoxycholic acid** | **Glyco-Lithocholic acid** |  |
| **Chenodeoxycholic acid-3S** | **Lithocholic acid-3S** |  |
| **Hyocholic acid** | **Tauro-Lithocholic acid-3S** |  |
| **Muricholic acid** | **Glyco-Lithocholic acid-3S** |  |
|  | **Hyodeoxycholic acid** |  |
|  | **Tauro-Hyodeoxycholic acid** |  |

3S suffix indicates sulfation on carbon 3

**Supplementary table 2: Indications for and outcomes of coronary angiograms**

| **Coronary artery disease** | |
| --- | --- |
| **Absent**  **(n=35)** | **Present**  **(n=45)** |
| **Indications** | |
| Stable angina: n = 26 | Stable angina: n = 34 |
| Left ventricular dysfunction: n = 4 | Left ventricular dysfunction: n = 1 |
| Preoperative: n= 3 | Preoperative: n= 3 |
| Positive stress test: n = 2 | Positive stress test: n = 7 |
| **Outcomes** | |
| Normal coronary vessels: n =35 | Non-occlusive atheromas: n = 13 |
|  | ≥50% stenosis  1 vessel: n = 18  2 vessels: n= 6  3 vessels: n = 8 |

**Supplementary table 3 : significant odds ratios associated with CAD**

|  | **Odd ratio (95% CI)** | ***P*** |
| --- | --- | --- |
| **Men versus women** | 6.29 (1.86, 21.32) | 0.003 |
| **Age, y** | 1.09 (1.03, 1.15) | 0.002 |
| **Total bile acids** | 0.51 (0.31, 0.85) | 0.01 |
| **Glyco-chenodeoxycholic acid** | 0.06 (0.01, 0.51) | 0.01 |

**Supplementary table 4**

**A. Concentrations of main species of bile acids in sera**

| **SERA** | **Coronary artery disease** | | ***P*** |
| --- | --- | --- | --- |
|  | **Absent** | **Present** |  |
| **CA** | 0.426 | 0.248 | **0.08** |
| **CDCA** | 0.974 | 0.391 | **<0.001** |
| **DCA** | 0.562 | 0.296 | **<0.05** |
| **LCA** | 0.041 | 0.014 | **<0.01** |
| **UDCA** | 0.148 | 0.070 | **<0.05** |
| **Conjugated** | 1.384 | 0.510 | **<0.001** |

Values are µmol/l

**B. Proportions of main species of bile acids in sera**

| **SERA** | **Coronary artery disease** | | ***p*** |
| --- | --- | --- | --- |
|  | **Absent** | **Present** |  |
| **CA** | 17 | 19 | ns |
| **CDCA** | 42 | 38 | ns |
| **DCA** | 32 | 34 | ns |
| **LCA** | 3 | 3 | ns |
| **UDCA** | 6 | 6 | ns |
| **Conjugated** | 67 | 59 | ns |

Values are percentages

**Supplementary table 5**

**A. Concentrations of main species of bile acids in feces**

| **FECES** | **Coronary artery disease** | | ***P*** |
| --- | --- | --- | --- |
|  | **Absent** | **Present** |  |
| **CA** | 318 | 368 | ns |
| **CDCA** | 313 | 419 | ns |
| **DCA** | 3602 | 4100 | ns |
| **LCA** | 2121 | 2382 | ns |
| **UDCA** | 242 | 121 | ns |
| **Conjugated** | 165 | 189 | ns |

Values are µmol/g of dried stool

**B. Proportions of main species of bile acids in feces**

| **FECES** | **Coronary artery disease** | | ***p*** |
| --- | --- | --- | --- |
|  | **Absent** | **Present** |  |
| **CA** | 4 | 5 | ns |
| **CDCA** | 5 | 5 | ns |
| **DCA** | 50 | 51 | ns |
| **LCA** | 36 | 33 | ns |
| **UDCA** | 2 | 2 | ns |
| **Conjugated** | 3 | 3 | ns |

Values are percentages

**Supplementary figure 1:** Standard bacterial alpha diversity indexes in the feces: Shannon, Simson, and Chao1 index

**Supplementary figure 2:** Comparison of 28 individual bile acids species concentration sera (free, glyco, tauro and sulfoconjugated). Unit : μmol/l

μmol/l

**
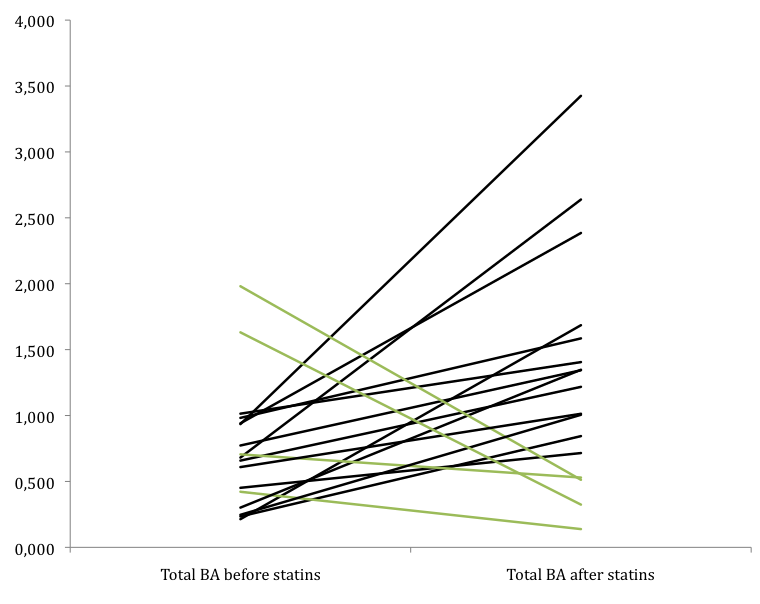
**

**Supplementary figure 3:** Individual progression of serum BA concentrations over 1 month of statin therapy

**Supplementary figure 4 :** Proposition of an hypothetical model summarizing causes and consequences of bile acids deficiency during coronary artery disease. The schematic representation resume the BA deficiency in CAD patients, the potential effect of statins, and the hypothesis of a decrease in hepatic "Spill Over" in CAD patients.

**
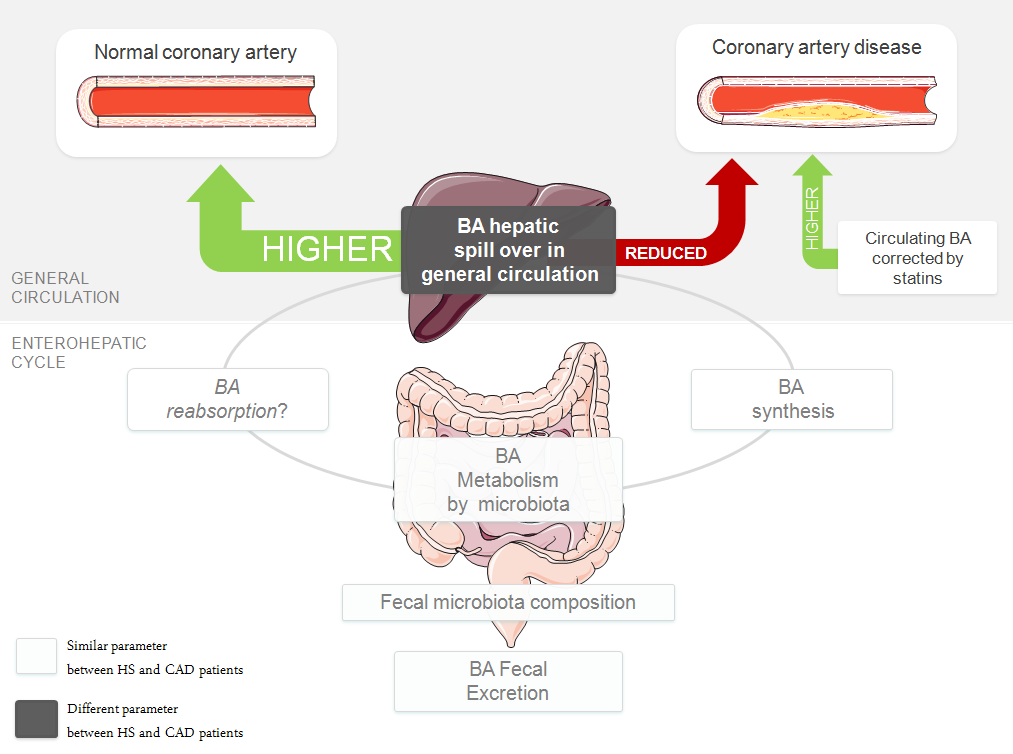
**

Supplementary methods:

**Bile acids and sera 7a-hydroxy-4-cholesten-3-one measurements in blood and feces**

- All chemicals and solvents were of the highest purity available. CA, deoxycholic acid (DCA), CDCA, ursodeoxycholic acid (UDCA), lithocholic acid (LCA), hyocholic acid and the corresponding glyco- and tauro-derivatives were obtained from Sigma-Aldrich (Saint-Quentin-Fallavier, France). The 3-sulphate derivatives were a generous gift from Dr J Goto (Niigata University of Pharmacy and Applied Life Sciences, Niigata, Japan) and the 23-nor-5 β-cholanoic acid-3α, 12α-diol was purchased from Steraloids, Inc (Newport, Rhode Island, USA). Because 3-sulpholithocholic acid is not commercially available, it was synthesised in our laboratory (ENS, Paris) using a previously described method (1), and further characterised by nuclear magnetic resonance and mass spectrometry. Acetic acid, ammonium carbonate and ammonium acetate were also purchased from Sigma-Aldrich (Saint-Quentin-Fallavier).

- Standard solutions : Standard stock solutions were prepared in methanol at a concentration

of 1 mg/ml and stored in a sealed container at −20°C. The stock solutions were pooled and diluted to obtain mixed-calibration BA solutions, ranging from 31.3 mg/ml to

31.3 ng/ml.

- Sample preparation Two microlitres of an internal standard solution (23-nor-5β-cholanoic acid-3α, 12α-diol at 1 mg/ml) was added to either serum (500 ml) or 0.1 g of faecal lyophilised samples using a Thermo Savant Speedvac (SPD 111V) coupled to a cooled vapour trap (RTV400)(2). For C4 quantification, 1 microliters of an internal standard solution (deuterated C4 form, D7C4, 7α-hydroxy-4-cholesten-3-one-d7, at 1 mg/ml) was added to the serum. The BAs were released from the binding protein by the addition of 0.4 M ammonium carbonate, at a concentration of 4 ml ammonium carbonate per 1 ml of sample, and incubated for 30 min at 60°C. For faecal samples, 2 ml of NaOH (0.1 M) was added and incubated for 1 h at 60°C before the addition of 4 ml of water.28 The solution was homogenised by two 30 s runs in an Ultra-Turrax disperser (IMLAB, Lille, France).

- The preanalysis cleanup procedure : a centrifugation of the sample (20 000×g for 20 min) as followed by solid-phase extraction using reversed-phase silica cartridges. These reversed-phase Chromabond C18 cartridges (100 mg; Macherey-Nagel, Düren, Germany) were preconditioned with 5 ml of methanol and 5 ml of water in succession. Samples were then loaded on the cartridge, and the subsequent elution steps were processed using a vacuum manifold. The cartridge was rinsed with water (20 ml), followed by hexane (10 ml), and then rinsed a second time with water (20 ml). The BAs were finally eluted and collected by methanol eluate. The methanol was evaporated under nitrogen at 50°C, and the residue was resuspended in 150 μl of methanol, 5 ml of which was injected into the high-performance liquid chromatography tandem mass spectrometry (HPLC MS/MS) system.

- HPLC MS/MS analysis. For the HPLC MS/MS analysis, the separation of BAs as a function

of polarity was accomplished using an analytical column (Pinnacle II C18, Restek, Lisses, France; 250 mm×3.2 mm (L×ID), 5 mm silica particle (Restek)) fitted on an HPLC binary

pump (Agilent 1100, Agilent Technologies France, Massy, France). The transfer line from the autosampler (Agilent) and column was maintained at 35°C. The 0.3–0.5 ml/min flow rate

was increased during the elution protocol. The mobile phase was composed of a mixture of ammonium acetate (15 mM, pH 5.3) and methanol. The HPLC was coupled in series with the

turbo ion-spray source of the tandem mass spectrometer (QTRAP 2000, Applied Biosystems/MDS SCIEX, Concord, Ontario, Canada). Electrospray ionisation was performed in the negative mode, with nitrogen as the nebuliser gas. The temperature of the evaporation gas was set at 400°C. The ion-spray, declustering and entrance potentials were set at −4500 V,

−60 V and −10 V, respectively. Collision-induced dissociation was achieved in a Q2 collision cell under various voltage potentials, depending on conjugation, and MS/MS detection was

operated with unit/unit resolution in the multiple-reactionmonitoring (MRM) mode. The dwell time of the ion trap was set at 70 ms for each transition.

- Data acquisition : the data were extracted using Analyst V.1.4.2 software. MRM at low collision energy focuses on transition reactions from precursor ions to product ions after the cleavage of taurine, glycine and sulphate fragments. For glycine-conjugated BAs, m/z 432, 448 and 464 were selected as the precursor ions, and m/z 74 was selected as the product ion. For taurineconjugated BAs, m/z 482, 498 and 514 were selected as the precursor ions, and m/z 80 was selected as the product ion. For sulpho-conjuguated BAs, the HSO4 sulphuric anion (m/z 97) from the sulphate moiety was selected as the product ion. For unconjugated BAs, m/z 375, 391 and 407 were selected as both the precursor and product ions, as no fragmentation could be identified at the low collision energy used. In addition, m/z 377 was selected for the internal standard (23-nor-5 β-cholanoic acid-3α, 12α-diol). The BA quantitation was expressed as the percentage of each specific BA (±SEM) out of the total BAs after calibration of the method, with weighed mixtures and normalisation relative to the internal standard (nordeoxycholic acid).

**Fecal DNA extraction and 16S rRNA gene sequencing**

Fecal DNA was extracted as described elsewhere by disruption (200-300mg of feces). Bacterial diversity was determined for each sample by targeting part of ribosomal genes. A 16S rRNA gene fragment comprising V3 and V4 hypervariable regions (16S (sense) 5’-TACGGRAGGCAGCAG-3’ and (antisense) 5’-CTACCNGGGTATCTAAT-3’) was amplified using an optimized and standardized 16S-amplicon-library preparation protocol (Metabiote®, GenoScreen, Lille, France) as described previously(3) . Briefly, 16S rRNA gene PCR was carried out using 5ng of genomic DNA according to Metabiote® protocol instructions using 192 bar-coded primers (Metabiote® MiSeq Primers, GenoScreen, Lille, France) at final concentrations of 0.2 μM and an annealing temperature of 50°C for 30 cycles. PCR products were cleaned up with Agencourt AMPure XP-PCR Purification system (Beckman Coulter, Brea, CA), quantified according to the manufacture’s protocol, and multiplexed at equal concentration. Sequencing was performed using a 300-bp paired-end sequencing protocol on the Illumina MiSeq platform (Illumina, San Diego, CA) at GenoScreen, Lille, France. Raw paired-end reads were subjected to the following process 1) quality filtering with the PRINSEQ-lite PERL script (4) by truncation of bases from the 3’end not with quality <30 based on the Phred algorithm, 2) paired-end read assembly using FLASH(5) (Fast length adjustment of short reads to improve genome assemblies) with a minimum length overlap of 30 bases and 97% overlap identity and 3) the search and removal of both forward and reverse primer sequences using CutAdapt, with no mismatches allowed in primers sequences. Assembled sequences for which perfect forward and reverse primers are not found were eliminated.

**16S rRNA genes sequence analysis**

Assembled sequences were analyzed using the Quantitative Insights Into Microbial Ecology (QIIME, version 1.8.0) software package (6). Sequences were assigned to Operational Taxonomic Units (OTUs) using the UCLUST algorithm (7) with 97% threshold of pairwise identity, and classified taxonomically using Greengenes reference database (8). Rarefaction was performed (20,000 sequences per sample) and used to compare abundances of OTUs across samples.

Principal component analyses (PCA) of Bray Curtis distance was built and used to assess the variation between experimental group. The number of observed species, the Shannon, the Simpson and the Chao1 diversity indexes were calculated using rarefied data (depth = 20,000 sequences/sample) and used to characterize species diversity in a community.

**Statistical analysis for microbiota analysis**

GraphPad Prism version 6.0 (San Diego, CA) was used for all analyses and graph preparation. For microbiota analysis, differential abundance analysis were performed using Linear Discriminant Analysis Effect Size (LEFSE) (9). Statistical significance of sample grouping for beta diversity analysis was performed using Permanova method (9999 permutations). Differences with a P value less than 0.05 were considered significant.

1. Tserng KY, Klein PD. Synthesis of sulfate esters of lithocholic acid, glycolithocholic acid, and taurolithocholic acid with sulfur trioxide-triethylamine. J Lipid Res. 1977 Jul;18(4):491–5.

2. Humbert L, Maubert MA, Wolf C, Duboc H, Mahé M, Farabos D, et al. Bile acid profiling in human biological samples: comparison of extraction procedures and application to normal and cholestatic patients. J Chromatogr B Analyt Technol Biomed Life Sci. 2012 Jun 15;899:135–45.

3. Liguori G, Lamas B, Richard ML, Brandi G, da Costa G, Hoffmann TW, et al. Fungal Dysbiosis in Mucosa-associated Microbiota of Crohn’s Disease Patients. J Crohns Colitis. 2016 Mar;10(3):296–305.

4. Schmieder R, Edwards R. Quality control and preprocessing of metagenomic datasets. Bioinformatics. 2011 Mar 15;27(6):863–4.

5. Magoč T, Salzberg SL. FLASH: fast length adjustment of short reads to improve genome assemblies. Bioinformatics. 2011 Nov 1;27(21):2957–63.

6. Caporaso JG, Kuczynski J, Stombaugh J, Bittinger K, Bushman FD, Costello EK, et al. QIIME allows analysis of high-throughput community sequencing data. Nat Methods. 2010 May;7(5):335–6.

7. Edgar RC. Search and clustering orders of magnitude faster than BLAST. Bioinformatics. 2010 Oct 1;26(19):2460–1.

8. McDonald D, Price MN, Goodrich J, Nawrocki EP, DeSantis TZ, Probst A, et al. An improved Greengenes taxonomy with explicit ranks for ecological and evolutionary analyses of bacteria and archaea. ISME J. 2012 Mar;6(3):610–8.

9. Segata N, Izard J, Waldron L, Gevers D, Miropolsky L, Garrett WS, et al. Metagenomic biomarker discovery and explanation. Genome Biol. 2011;12(6):R60.
